# Sensor-Based Technology for Social Information Processing in Autism: A Review

**DOI:** 10.3390/s19214787

**Published:** 2019-11-04

**Authors:** Andrea E. Kowallik, Stefan R. Schweinberger

**Affiliations:** 1 Early Support and Counselling Center Jena, Herbert Feuchte Stiftungsverbund, 07743 Jena, Germany; andrea.kowallik@uni-jena.de; 2 Social Potential in Autism Research Unit, Friedrich Schiller University, 07743 Jena, Germany; 3 Department of General Psychology and Cognitive Neuroscience, Friedrich Schiller University Jena, Am Steiger 3/Haus 1, 07743 Jena, Germany; 4 Michael Stifel Center Jena for Data-Driven and Simulation Science, Friedrich Schiller University, 07743 Jena, Germany; 5 Swiss Center for Affective Science, University of Geneva, 1202 Geneva, Switzerland

**Keywords:** automatic recognition, face, voice, body motion, autism spectrum disorder (ASD), assessment, intervention

## Abstract

The prevalence of autism spectrum disorders (ASD) has increased strongly over the past decades, and so has the demand for adequate behavioral assessment and support for persons affected by ASD. Here we provide a review on original research that used sensor technology for an objective assessment of social behavior, either with the aim to assist the assessment of autism or with the aim to use this technology for intervention and support of people with autism. Considering rapid technological progress, we focus (1) on studies published within the last 10 years (2009–2019), (2) on contact- and irritation-free sensor technology that does not constrain natural movement and interaction, and (3) on sensory input from the face, the voice, or body movements. We conclude that sensor technology has already demonstrated its great potential for improving both behavioral assessment and interventions in autism spectrum disorders. We also discuss selected examples for recent theoretical questions related to the understanding of psychological changes and potentials in autism. In addition to its applied potential, we argue that sensor technology—when implemented by appropriate interdisciplinary teams—may even contribute to such theoretical issues in understanding autism.

## 1. Introduction

Throughout the last decades, the number of people diagnosed with an Autism Spectrum Disorder (ASD) increased dramatically [1,2] and so did the need for high-quality diagnostic protocols and therapies. With the ongoing progress in computer sciences and hardware, a lot of creative ideas emerged on how to use sensor data to identify and observe autistic markers, support diagnostic procedures and enhance specific therapies to improve individuals’ outcomes.

ASD is a behaviorally defined group of neurodevelopmental disorders that are specified by impaired reciprocal social communication and restricted, repetitive patterns of behavior or activities (DSM-5), [3]. The symptoms are usually apparent from early childhood and tend to persist throughout life [4]. Common social impairments include a lack of social attention as evident in abnormal eye gaze or eye contact [5] and social reciprocity such as in reduced sharing of emotions in facial [6] or vocal behavior [7]. Further, only a minority of the affected people report having mutual friendships [8]. Related to the restricted and repetitive behaviors, stereotyped motor movements and speech are the stand-out features in many people with ASD [9]. Other symptoms are insisting on sameness and routines [10], special interests and hyper- or hyporeactivity to sensory input from various modalities [11]. The exact profile and severity of symptoms in people with ASD as well as their personal strengths and coping capabilities vary to a great degree, and so does their need for support.

Reasons for the increased prevalence over the last decades include a more formalized diagnostic approach and heightened awareness. The current ‘gold standard’ for a diagnosis of ASD consists of an assessment of current behavior, a biographical anamnesis, and a parental report, all collected and evaluated by a trained multi-professional team [12]. Although the screening and diagnostic methods for ASD improved throughout the last years, many affected people, especially women [13] and high functioning people, still receive a late diagnosis. Since early interventions have been shown to be most effective for improving adaptive behavior, as well as IQ and language skills [14], there is continued demand for methods promoting early assessment in order to avoid follow-up problems. In this context, progress in automatic and sensor-assisted identification of ASD-specific behavioral patterns could make an important contribution to an earlier and less biased diagnosis.

Even beyond assessment, advances in digital technology are highly relevant for autism, and in more than just one way. First, there is some evidence that many autistic people show behavioral tendencies to interact with technology and to potentially prefer such interactions to interactions with humans. It is thought that autistic traits are related to systemizing, the drive to analyze how systems work, as well as to predict, control and construct systems [15]. In this context, high information technology (IT) employment rates are often used as a proxy for higher rates of strong systemizers in a population. Intriguingly, recent research from the Netherlands reported that the prevalence of childhood autism diagnoses, but not of two control neurodevelopmental diagnoses (i.e., ADHD and dyspraxia), was substantially higher in Eindhoven, a classical (IT) region, when compared to two control regions (Utrecht and Haarlem) that had been selected for high demographic and socio-economic similarity in criteria other than the proportion of IT-related jobs [16]. Second, and qualifying any simplistic interpretation of this correlative (but not necessarily causal) relationship, there is evidence that technology can potentially provide powerful social support for children with autism. For instance, children with ASD often perform better with a social robot than a human partner (e.g., in terms of enhanced levels of social behavior towards robots), tend to perceive interactions with robots as positive [17], and subsequently show reduced levels of repetitive or stereotyped actions. For a recent review, see Pennisi et al. [18].

Scientific interest in the utilization of sensor technology to gain an understanding of people with ASD has increased considerably in the recent past. Some fields of research focus on different neurobiological assessments and try to identify autism-specific signals or ‘biomarkers’ to better understand the neurobiological underpinnings of the disorder. Good overviews covering methods including electroencephalography (EEG), magnetoencephalography (MEG), and functional magnetic resonance imaging (fMRI) are provided by Billeci et al. [19] and by Marco et al. [20]. Other research focused on an autonomic activity such as heart rate variability (HRV) or skin conductance responses (SCR). These can be studied with Wearable devices, typically in the context of emotional monitoring in ASD as seen in a review by Taj-Eldin, et al. [21]. Applications in VR environments [22,23,24] have also been reviewed as promising methods to train and practice social skills.

The aim of this review is to provide an overview of the current state of research using sensor-based technology in the context of ASD. We focus on sensor technology that is applicable without constraining natural movement, and on sensory input from the face, the voice, or body movements. Accordingly, this review does not consider evidence from wearable technology, VR, or psychophysiological and neurophysiological recordings. Note also that while we provide details of our procedure for identifying relevant original findings to enhance reproducibility, this paper represents a thematic review in which we pre-selected for contents as described below, and in which we occasionally discuss additional relevant findings that were not formally identified by this literature search. For instance, we may refer to some key findings regarding psychological theories of human social or emotional communication where relevant, even when the findings were not obtained with individuals with autism.

## 2. Literature Search

We performed parallel literature searches on Web of Science, PsychInfo, PubMed, and IEEE Xplore. Considering the rapid advance of technology throughout the last decades, we focussed on the last 10 years (2009–2019) to give an overview of recent developments in this field. Searches were performed on 29 May 2019. Our search terms were (autis* OR ASD OR Asperger) AND (sensor OR sensors) AND (fac* OR voice* OR body motion* OR person* OR regoc* OR identi* OR emotio* OR diagnos*). Furthermore, the considered language was restricted to English. Overall, the search resulted in 386 articles (Web of Science *N* = 185, IEEE Explore *N* = 109, PubMed *N* = 52, PsychInfo *N* = 40). Note that we only included publications with original data from an ASD or high-risk group, such that new algorithms on preexisting data sets were not included. We also included the reference lists of identified articles with respect to additional screening for relevant publications. After removing duplicates, this selection process resulted in a total of 36 articles. These are discussed below, and those articles that focused on assessment or intervention are additionally summarized in Tables 1 and 2, respectively.

## 3. Supporting Assessment: Identification of ASD Related Features

### 3.1. Facial Information

#### 3.1.1. Facial Movements

For facial movements, researchers tended to focus on emotional facial information, following the idea that impaired social communication in ASD can be framed as deficits in emotional communication regarding both perception and expression [25], and the finding that people with ASD may show reduced or idiosyncratic emotional expressions [6,26]. Against this context, it may be useful to keep in mind that ASD-specific impairments in face perception are not restricted to emotional expressions, but also affect other aspects such as facial identity [27]. Similarly, expression in social communication may be affected in subtle ways that go beyond emotional expressions [28]. The original articles on sensor-based assessment in terms of identification of autism spectrum disorders (ASD)-related features, regarding facial movements and other forms, represented in this review are listed in Table 1.

Samad et al. [29] compared facial expressions from an ASD and a typically developing (TD) group, with 8 participants each. Computing facial curvatures from 3D point cloud data, they found equally intense but more asymmetrical facial expressions in the ASD group. Another study [30] used a single webcam mounted on a TV screen to record toddlers’ spontaneous facial expressions when confronted with emotional cartoons. Comparing descriptive data from small groups with five children each, they reported that the lower face seemed to be more important in distinguishing between the ASD and TD groups. Leo et al. [31] presented a new processing pipeline on 2D video data that aimed at assessing facial expressions in ASD children specifically. They estimated the production skills for individual children based on verbal instructions to express these emotions, separately for individual face parts and emotions. The performance of 17 boys with ASD was variable, and some boys exhibited differential production scores for different categories of emotions. Note that this finding is in broad agreement with theories that emphasize category-specific mechanisms, and with componential approaches to emotion [32,33]. Samad et al. [34] used a storytelling avatar, also with the aim of eliciting spontaneous emotions to differentiate between ASD and TD groups with 10 participants each. Comparing 3D data on the level of facial action units (AUs) they found overall lower AU activations in the ASD group and lower correlations between the AUs. The deviant activation in AUs 6, 12, and 15 (cheek raiser, lip corner puller, and lip corner depressor, respectively), were found to be promising markers for ASD. As a more serious screening approach, the App ‘Autism & Beyond’ [35] recorded toddlers’ facial responses to certain stimuli with the front camera and classified them into positive, neutral, or negative. Using a large database of 1756 children, the associated facial expressions, along with eye gaze and parental reports, with Autism Spectrum risk status. Comparisons between high- and low-risk groups revealed that high risk for ASD was associated with higher frequencies of neutral facial expressions, and with lower frequencies of positive expressions.

#### 3.1.2. Eye Gaze

One major domain in which autistic people are frequently described to behave unusually relates to oculomotor behavior, including low levels of eye contact during communication, and low levels of directional signaling via eye gaze. In fact, a current target article challenges the common belief that autistic people lack social interest in others, and suggests that this interpretation could be erroneously elicited by unusual behavior such as low levels of eye contact [36]. While this may be a useful context to keep in mind when reading this section, there is agreement that the assessment of eye gaze can help to identify behavioral patterns that are relevant for autism [37].

Chawarska and Shic [38] compared toddlers with ASD and TD of different age groups (2 and 4 years old) with an eye-tracking system while they watched neutral faces. Although all groups spent equal time looking at the screen, a restricted scanning pattern in the ASD group was found, with relative neglect of the mouth area. Additionally, the older ASD group spent less time looking at inner facial features in general, even compared to the younger ASD group. According to the authors, this indicates a different scanning pattern of children with ASD that emerges throughout early childhood and suggests less looking at the mouth as the best early predictor for ASD. Liu et al. [39] used a machine-learning framework on eye-tracking data of 29 ASD vs. 29 TD children (mean age 7.90 years) looking at images of faces embedded in a learning task with repeated presentations. Their proposed framework was able to identify ASD children both from age- (mean age 7.86 years) and IQ-matched (mean age 5.74 years) control groups with high classification accuracy (up to 88.5%). Król and Król [40] used eye-tracking to study the effect of including temporal information into spatial eye-tracking of face stimuli, thus creating scan paths for 21 ASD and 23 TD individuals (mean age around 16 years). They found a difference in face-scanning not only in spatial properties but also in temporal aspects of eye gaze, even within short exposures (about 2 s) to a facial image. Classification of group membership based on a machine-learning algorithm on spatial and temporal data led to better accuracy (55.5%) than classification based on spatial data alone (53.9%), although overall accuracy was rather low. Note that discrepancies between classification accuracies in this study and the study by Liu et al. [39] need to be seen in the context of specific conditions of each study, and could potentially be attributed to the facial stimuli and trial numbers used, the experimental task instructions, methodological differences in data analysis, or differences in the samples tested.

In a more general study on the visual scanning of natural scene images, Wang et al. [41] compared an ASD and a TD group (*N* = 20 and 19, respectively) at different levels of perception. The ASD group was found to fixate more towards the center of an image (pixel level), less on objects in general (object level) and less on certain objects (e.g., faces or objects indicated by social gaze), but more on manipulatable objects (semantic level).

Overall, facial data suggest that facial movements, either in response to emotional stimuli or as an imitation of seen facial expressions, comprise relevant features that may be markers for autism. Recordings of spontaneous emotional responses could be of particular benefit when assessing small children or nonverbal people, although the importance of age-appropriate task instructions should be considered. Across several studies, eye gaze data reveal different scanning patterns for people with ASD, particularly when viewing faces. However, we believe that further systematic research into fixation patterns and scan paths for more complex natural scenes could enrich our current understanding with additional insights. Similarly, the observation of reduced eye contact/mutual gaze in ASD, reviewed in Jaiswal et al. [50], points at a technologically challenging but theoretically relevant field of future investigations using sensor technology.

### 3.2. Voices

A scientific metaphor that has become somewhat popular describes the voice as an “auditory face” [55], emphasizing the fact that voices, just like faces, provide rich information about a person ´s emotions, but also about identity, gender, socioeconomic or regional background, or age (for review, see [56]). In the context of autism, and like for faces, researchers focused on deficits in vocal emotional communication and pointed out that these deficits tend to affect multiple modalities including voices, faces, and body movement [57]. At the same time, deficits in vocal communication may also affect other aspects such as vocal identity perception [58,59], and vocal expression of autistic people in communication could be affected beyond emotional expressions. 

One path for detecting auditory markers for autism in voices is linked to the symptom of repetitive behaviors or ‘vocal stereotypies’. One study conducted a subspace analysis from acoustic data of autistic children and reported a good detection of vocalized non-word sounds [42]. Subsequently, Min and Fetzner [43] also used subspace learning for vocal stereotypies and trained dictionaries to differentiate between vocal stimming (a nonverbal vocalization often observed in autism) and other noises. Using a small sample of 4 children with ASD who lacked verbal communication (age not reported), the authors could e.g., detect vocal stimming and predict perceived frustration reasonably well, although the study was regarded as preliminary. For verbal children, other potential vocal markers could include prosody. Marchi et al. [44] created an evaluation database in three languages with ASD and TD children’s emotionally toned voices which were analyzed using the COMPARE feature set. Groups comprised between 7 and 11 individuals per language, and children were between 5 and 11 years old. Comparisons between groups and emotions showed relatively poor classification performance ASD children’s’ voices, particularly for ‘Anger’ for both the Swedish and English dataset, and for ‘Afraid’ for Hebrew-speaking children with ASD. Although using a pre-existing dataset, a study on automatic voice perception showed that an algorithm successfully classified up to 61.1% of voice samples of children actually diagnosed with and without autism [60]. Ringeval et al. [45] assessed verbal prosody in ASD children, children with pervasive developmental disorder (PDD), children with specific language impairment (SLI), and TD children (aged around 9–10 years, with 10–13 children per clinical group). Specifically, these authors recorded performance during an imitation task for sentences with different intonations (e.g., rising, falling). The rising intonation condition was reported to best discriminate between groups, and the authors interpreted their findings as indicating a pronounced pragmatic impairment to prosodic intonation in ASD.

When compared with facial data, the use of sensors for automatic analysis of markers for autism in voices clearly is still at an early and preliminary stage. The studies reviewed above that use original data typically rely on small data sets from few people. Although initial findings suggest that the systematic search for vocal markers of autistic traits could be highly promising, more research is clearly warranted at this stage. When considering that similar impairments in facial and vocal emotional processing could be present in ASD [57], multisensory assessment of emotional behavior in future studies could be particularly promising.

### 3.3. Body Movement

The identification of ASD-associated movement patterns has been the subject of intense research, primarily focusing data from accelerometer sensors [42,61]. As the display of stereotypical body movement patterns is a core symptom of ASD, it has been a target feature in computer vision. Gonçalves et al. [46] used a simple gesture recognition algorithm on 3D visual data automatically detecting hand-flapping movements. Validation of these data with 2D video data suggested that automatic ‘hand flapping’ detection delivers valuable information for monitoring autistic children, as in the case of special needs schools. Jazouli et al. [47] also used the same sensor but based their analysis on a $P Point-Cloud Recogniser to automatically detect body rocking, hand flapping, fingers flapping, hand on the face and hands behind back with an overall mean accuracy of 94%.

Rynkiewicz et al. [47] studied the role of non-verbal communication in the setting of an ADOS-2 assessment for children aged 5–10. They used a 3D sensor for automatic gesture analysis of the upper body, while the boys (*N* = 17) and girls (*N* = 16) with high-functioning ASD performed two assessment-related tasks. Females had a higher gesture index than boys although they had less verbal communication skills and a more impaired ability to read mental states from faces. The authors suggested that the vivid use of gestures in girls (generally less common in our current understanding of the autistic phenotype) may contribute to possible under-diagnosis of autism in females. This might further lead to the more general use of automatic gesture analysis that is currently performed by professional human raters in the course of these assessments. A study by Anzulewicz et al. [48] used the touch and inertial sensors of a tablet to assess the specific movement patterns of children with autism (*N* = 37) and an age- and gender-matched TD group (*N* = 45) while playing simple games. Apart from interesting findings including the use of greater force, larger and more distal gestures and faster screen taps in the ASD group, they also tested different machine learning algorithms to classify between groups, with promising results.

In summary, the use of 3D data has been preferred by researchers investigating body movements. While it seems to be possible to detect certain stereotypical movements, there is still a lack of large-scale studies. However, even coarser movement indices (including, for instance, gesture indices or general movement patterns) may also provide meaningful information for the identification and differentiation of autistic behavioral markers.

### 3.4. Multimodal Information

Multimodal approaches to automatic analysis of behavior in ASD are still infrequent. Samad et al. [49] used facial motion, eye-gaze and hand movement analysis of adolescents with (*N* = 8) and without ASD (*N* = 8) on tasks involving 3D expressive faces. They found a reduced synchronization of facial expression and visual engagement with the stimuli in the ASD group, as well as poorer correlations in eye-gaze and hand movements. Jaiswal et al. [50] designed an automatic approach based on 3D data of facial features and head movements that detected ASD (vs. TD and vs. comorbid ASD and ADHD, with *N* between 11 and 22 per group) in adults with high accuracy in classification performance. Their recorded data consisted of participants reading, listening to and answering to questions taken from the ‘Strange Stories’ task [62] often used in ASD-assessments.

Overall, the integration of multimodal information, unfortunately, is not yet common in this field, despite the fact that there are reasons to expect that multimodal assessments will provide ample additional information (for instance, on synchronization or complementarity of signals from different channels). Thus, it can be expected that future use of multimodal assessments has the potential to substantially improve both the identification of markers for autistic traits and classification results.

### 3.5. Cognition and Social Behavior

A substantial body of research has suggested that autism is characterized by changes in cognitive information processing. For instance, autistic people may have difficulties in cognitively simulating the observed actions of communication partners – a process that has been related to the so-called human mirror neuron system, and that has been inferred via neurophysiological recordings [63]. Via experiments with social behavioral assessments, autism has also been related to a deficit in forming a theory of mind about other people [64]. Researchers in this field have long focused on inferences about “core” cognitive deficits from observational data, even though results can be very inconsistent across situations or studies [65,66]. We also welcome an increasing awareness of the danger that researchers wrongly infer putative core deficits by misinterpreting observational data [36]. Of course, inferences about core cognitive processes most typically need to be made from observational information. It is this context in which we foresee that sensor data, beyond their immediate value for an individual study, might eventually contribute to resolving theoretical controversies. One of these is the degree to which we should frame cognitive changes in autism in terms of general “core” deficits, or rather in terms of domain-specific deficits that emerge in a concrete perceptual and interactional context.

The automatic analysis of playing behavior could be a promising screening tool to identify stages of development, developmental delays and specific forms of deficits. In a preliminary study with only a single completely analyzed data set of a TD child [51], play data from smart toys with embedded acceleration sensors, as well as simultaneous video and audio recordings, were automatically characterized as certain forms of play behavior (exploratory, relational, functional), roughly indicating a certain stage of development. While this approach seems interesting, current limitations include the verbal prompting nature of their setting, which only allows children with good verbal skills to be assessed.

Joint attention is a basic communicational skill of sharing attention between communicational agents towards an object and has long been considered to be a precursor of a theory of mind [67] which may be reduced in people with ASD. In a study investigating a robot-assisted joint attention task [52] in children with and without ASD (*N* = 16 each), they captured participants’ orientation using 3D sensors over a timespan. In addition to findings of significantly less joint attention in the ASD group’s interaction with the robot, the ASD group also showed decreased micro-stability in the trunk area that was tentatively interpreted as a consequence of increased cognitive cost. In another study [53], attentional and orienting responses to name calls were studied in toddlers, aged 16–31 months, with ASD (*N* = 22) and TD (*N* = 82). Achieving a high intra-class-correlation with human raters, automated coding offered a reliable method to detect the differential social behavior of toddlers with ASD, who responded to name calls less often and with longer latency. Petric et al. [54] designed and further developed a robot-assisted subset of the ADOS assessment. They included certain social tasks (name-calling, joint attention) to assess information on eye gaze, gestures, and vocal utterances. The agreement of the robot’s classifier and clinicians’ judgments were evaluated as promising, but the results should be regarded with caution given the very small sample of children (ASD *N* = 3, TD N = 1).

Overall, while researchers have begun to assess joint attention in ASD, sensor-based assessment of other functional domains of cognition and social behavior still awaits systematic research. In particular, research on a few putative “core” areas of social cognition, including on observational tasks that probe theory of mind in ASD, is currently lacking.

## 4. Supporting Interventions

While accurate early diagnosis and an assessment of specific impairments are crucial, they also are prerequisites that inform environmental adjustment, intervention, and training approaches which ultimately can be valuable for the individual person with ASD following diagnosis. Technically-assisted training often has the benefit of being readily available, problem-specific, cost-effective, and widely accepted by affected children. Additionally, smart responses of training systems that give reliable, immediate feedback and appraisal can be highly beneficial for fast learning results. Note that although we identified many publications on interventions aiming at autism as a target condition, many of these reported conceptual or technological contributions and few of them presented original data from people with ASD that qualified them for inclusion in this review (cf. Section 2). The original articles presented in this review, regarding sensor-based supporting interventions for ASD, are listed in Table 2.

### 4.1. Emotion Expression and Recognition

Emotion expression, especially from the face, is considered highly relevant in autism research. A game called FaceMaze [68], in combination with automatic online expression recognition of the user was specifically developed to improve facial expression production. In this game, children played a maze game while posing ‘happy’ or ‘angry’ facial expressions to overcome obstacles in the game. In a pre-post-rating with naive human raters, quality ratings for both trained expressions (happy and angry expression) in ASD children (*N* = 17, aged 6–18) increased in the post-test while ratings for an untrained emotion (surprise) did not change. Another smaller study created a robot-child-interaction and tested it with three children with ASD that were to imitate a robot’s facial expression [69]. The robot correctly recognized the children’s’ imitated expressions through an embedded camera in half of the cases. In these cases, it was able to give immediate positive feedback. It also correctly did not respond in about one-third of the trials where there was no imitation by the participants. Piana, et al. [70] designed a serious game with online 3D data acquisition that trained children with ASD (*N* = 10) in several sessions to recognize and express emotional body-movements. Both emotion expression (mean accuracy gain = 21%) and recognition (mean accuracy gain = 28%) increased throughout the sessions. Interestingly, performance in the T.E.C. (Test of Emotion Comprehension, assessing emotional understanding more generally), increased as well (mean gain = 14%).

### 4.2. Social Skills

Robins et al. [71] created an interactive robot (KASPAR) with force sensitive resistor sensors. They later planned to use KASPAR for robot-assisted play to teach touch, joint attention and body awareness [72], although conclusive data on experimental results from interactions between individuals with autism and KASPAR may still be in the pipeline. Learning social skills also presupposes attention to potential social cues and social engagement. Costa et al. [73] reported preliminary research on using the LEGO mindstorm robots with adolescents with ASD (*N* = 2), in an attempt to increase openness and induce communication since the participants actively had to provide verbal commands or instructional acts. They reported that the two participants behaved differently, one being indifferent, and one being increasingly interested in the interaction. Wong and Zhong [74] used a robotic platform (polar bear) to teach children with ASD (*N* = 8) social skills. They found, that within five sessions an increase in turn-taking, joint attention and eye contact was observable, resulting in overall 90% achievement of individually defined goals.

Greeting is a basic element of communication. In a greeting game with 3D body movement as well as voice acquisition [75], a participant would play an avatar with his own face, learning to greet (vocalization, eye contact and waving) and get immediate appraisal upon success. A single case study suggested that this intervention can be effective at teaching greeting behavior. As a more complex pilot intervention, Mower et al. [76] created the embodied conversational agent ‘Rachel’ that acted as an emotional coach in guiding children through emotional problem-solving tasks. Of their two participants with ASD, audio and video data were acquired for post hoc analysis and tentatively suggested that the interface could elicit interactive behavior.

Overall, there is some evidence that sensor technology can improve social skills in people with autism, and the use of sophisticated robotic platforms can be regarded as particularly promising. As limitations, it needs to be noted that all studies that met the criteria to be included in this review only tested very few participants, and that there typically were no real-world follow-up tests reported. As a result, a systematic quantitative assessment of treatment effects and effect sizes, as well as a comparison with more conventional interventions (e.g., social competence training) will require substantial cross-disciplinary research. Moreover, most studies were driven by a combination of theoretically interesting and technically advanced approaches, and from the perspective of typical development. Designing more user-centered and irritation-free approaches could promote both usability and motivation for people with autism to engage in technology-driven interventions.

## 5. Monitoring

Monitoring a child’s emotional state or behavioral changes can be crucial for the outcomes of a learning environment. As discussed above, emotional expressions from people with ASD may differ in several respects from those of TD people. As a result, there is a higher risk that caregivers or interaction partners overlook or misinterpret the emotional state of people with autism.

Del Coco et al. [77] created a humanoid and tablet-assisted therapy setup that was trained to monitor behavioral change in children with ASD via a video processing module. Besides creating a visual output of behavioral cues, they computed a score for affective engagement (happiness related features) from visual cues such as facial AUs, head pose and gaze that provides the practitioner with a behavioral trend along with the treatment. Dawood et al. [78] used facial expressions, eye gaze and head movements to identify five discrete emotional states of young adults with ASD in learning situations (e.g., anxiety, engagement, uncertainty). Their resulting model yielded a high validity in identifying emotional states of participants with high-functional ASD. At the same time, a lower validity was found for TD participants, suggesting differential facial expressions of certain emotional states in ASD. For monitoring social interactions, Winoto et al. [79] created a machine-learning-based social interaction coding of 3D data around a target user. Kolakowska et al. [80] approached automatic progress recognition with different tablet games. Over a 6-month time window, they were able to identify movement patterns in their study group of children with ASD (*N* = 40), that not only related to development in fine motor skills but also other fields like communication and socio-emotional skills. Overall, these initial studies suggest that sensor-based monitoring of emotional and behavioral changes may support caregivers in optimizing learning outcomes. 

## 6. Discussion

The studies discussed above demonstrate substantial research activities towards using sensor-based technology in the context of autism overall, with attention to multiple aspects including diagnosis/classification and intervention. At the same time, it appears that much current research is largely driven by fast technological progress in terms of innovative engineering and data analysis methods. It remains a significant challenge to reconcile these developments with the specific testing of psychological or neuroscientific theories regarding functional changes and potentials in autism. Similarly, systematic studies with theory-driven protocols and larger samples are required to evaluate in more detail both the diagnostic and interventional potential of sensor-based technology. For the ultimate goal of evaluating its practical relevance, quantitative assessments of diagnostic sensitivities and specificities, or of treatment effect sizes, will be as important as will be comparative studies with more traditional approaches to diagnosis and intervention.

One of many examples of how sensor technology has the potential to go beyond application, and to contribute to current neurocognitive theories of communication is related to the theory of a tight link between perception and motor action in communication. This link now has been firmly established in speech communication [81], but there are reasons to believe that perception and action are also closely linked in nonverbal emotional and social communication. For instance, listening to laughter normally activates premotor and primary motor cortex [82], and may involuntarily elicit orofacial responses in a perceiver in parallel. In turn, there also is initial evidence that voluntary motor imitation can actually facilitate facial emotion recognition, particularly in people with high levels of autistic traits [83] who are thought to engage less in spontaneous imitation. A consistent theoretical account for such findings is that imitation, and covert sensorimotor simulation of others’ actions, may be based in part on the so-called mirror neuron system. This system consists of neurons that fire not only when a person performs an action, but also when s/he observes the same action in another individual. However, the human mirror neuron system is thought be specifically impaired in autism [63], and a subset of promising intervention approaches for autism using neurofeedback [84] are based on this theory. However, it should be noted that the underlying theory remains disputed [85].

Findings such as those by Lewis and Dunn [83] may be taken to suggest that interventions that promote facial imitation of emotions in autistic people should also support their abilities for emotion recognition and bidirectional communication. However, it is technically challenging to objectively quantify the degree of facial imitation, and in fact, a limitation of the study by Lewis and Dunn was that these authors failed to quantify imitation beyond simply asking participants to rate their own degree of imitation. Other studies measured facial imitation more objectively but typically did so by measuring the facial muscle response for selected target action units with electromyography (EMG, e.g., [86,87]). Although this can provide an objective measure of facial imitation, the fact that the method uses recording electrodes attached to facial muscles has many drawbacks. For instance, one concern is that this technology could draw the participants’ attention to their own facial behavior, which in turn could influence facial action. We believe that contact- and irritation-free assessment of imitation as provided by modern sensor and real-time facial emotion recognition technologies is the method of choice to promote better understanding not only the role of spontaneous facial imitation in emotion recognition in normal communication, but also to determine the potential role of impaired links between perception and action for communication difficulties in people with autism.

While the research discussed in this review appears to underline a great potential for the use of sensor technology, in particular in the context of autism, it is equally clear that many current tests of assessments or interventions will benefit in validity from a clear conceptual framework of autism spectrum disorders in the developmental perspective. At present, and honoring findings of large individual variability within both people with ASD and TD, results that were obtained with only a few participants (not always well described, and sometimes obtained in the absence of a TD group) or with experimental groups that are not comparable with respect to their basic characteristics (e.g., age, gender, IQ) need to be interpreted with caution in order to avoid biased or overgeneralized interpretation of individual study findings.

Other potential obstacles relate to sophisticated developments (and costs) of some of the systems used, which make them unlikely to become available in greater quantities. Moreover, even readily available systems may get discontinued or run out of support, such as in the case of Microsoft’s Kinect in 2017, and this provides great challenges for large-sample research in autism which often takes years to complete. Research aiming at training and modeling behavior of people with ASD also will increasingly need to consider usability, to the extent that the relevant systems are to be used by individuals with ASD, their parents, caregivers, and therapists.

Finally, compared to the typical approach of developing sensor-based technology with neurotypical individuals before applying it to people with autism, a more promising strategy may be one in which technology design originates from a user-centered perspective, with autistic people as users actively involved in the process. Such an approach has been forcefully advocated by Rajendran [88], who argues that this may both enhance our understanding of autism and promote better inclusivity of people with autism in an increasingly digital world. At the same time, such technologies ultimately can be useful for people without autism as well. This is because autism is seen as a unique window into social communication and social learning more generally.

## 7. Conclusions

Technical advancements and the ongoing developments in sensor technology and data science promise to unlock huge potentials for the diagnosis and understanding of autism, and for supporting affected people with training or intervention programs that can be tailored to their specific needs. At the same time, living up to these potentials calls for a concerted and interdisciplinary effort in which computer scientists, engineers, psychologists, and neuroscientists jointly collaborate in large-scale research projects that can uncover, in a quantitative manner, the efficiency of these approaches. In our view, this will be the route not only for establishing routine contributions to evidence-based diagnosis and interventions in autism [89] but also to ensure that more people with autism can genuinely benefit from tailor-made technology.

## Figures and Tables

**Table 1:** Original Articles on Sensor-Based Assessment in Terms of Identification of autism spectrum disorders (ASD)-related features.

Domain of Behavior	Reference	Solution Name	Sensor/Parameters	Sample (Mean Age)	Setting and Stimuli	Results
Facial Movements	Samad et al., 2016 [29]	Sony EVID70 Color Camera, 3dMD	2D, 3D Imaging	ASD: 8 (13 y), TD: 8 (16 y)	Emotion Recognition Task: 12 3D Faces	ASD: Intense, Asymmetrical Facial Expressions with Lack of Differential Facial Muscle Actions
Facial Movements	Del Coco et al., 2017 [30]	Webcam	2D Imaging	ASD: 5 (5 y), TD: 5 (age-, Gender-Matched)	Watching 9 Emotion Eliciting Videos Taken from Famous Cartoons	ASD Descriptively Exhibited Less Facial Behavioral Complexity; Lower Face Seemed More Significant Than Upper Face to Distinguish Between TD-ASD
Facial Movements	Leo et al., 2018 [31]	Off-The-Shelf Camera	2D Imaging	ASD: 17 (9 y)	Emotion Production Task: 4 Expressions (Happiness, Sadness, Fear, Anger)	Descriptive Scores for Upper and Lower Face Parts
Facial Movements	Egger et al., 2018 [35]	iPhone	2D Imaging (Front Camera)	High Risk: 555 (3 y), Low Risk: 1199 (3 y)	Watching Video Clips	More Neutral and Less Positive Emotional Reactions in High-Risk Group
Facial Movements	Samad et al., 2019 [34]	PrimeSense	3D Imaging	ASD: 10 (14 y), TD: 10 (13 y)	Watching Story Content Narrated by The Animated Avatar	ASD: Overall Lower FAU Activation, Higher Activations of FAU 15, Limited Activation of FAUs In Response to Encountering Negative Emotional States; Concurrent Activations of Several FAU Pairs Are Found to Be Absent
Eye Gaze	Chawarska and Shic, 2009 [38]	iView X™ RED	Eye-Tracking	ASD: 44, TD: 30	Watching Color Images of Affectively Neutral Female Faces	ASD: Attended to Visual Scenes Containing Faces to A Similar Extent; ASD Look Less at Inner Facial Features, Older ASD Spent Less Time with Inner Features Than Younger, ASD Spent Less Time Looking at the Mouth
Eye Gaze	Liu et al., 2016 [39]	Tobii T60	Eye-Tracking	ASD: 29 (8 y), TD-Age: 29 (8 y), TD-Ability: 29 (6 y)	Face Memorization and Recognition Task	Classification Accuracy Predicting ASD: 88.51% (p < 001)
Eye Gaze	Król and Król, 2019 [40]	SMI RED250	Eye-Tracking	ASD: 21 (16 y), TD: 23 (16 y)	Face Perception Tasks with 60 Color Photographs of Faces (FACES Database)	Prediction ASD vs. TD Group Membership: Based on Only “Spatial” Information (M = 53.9%) Was Significantly Smaller Than That of the “Spatial + Temporal” Model (M = 55.5%)
Eye Gaze	Wang et al., 2015 [41]	Tobii X300	Eye-Tracking	ASD: 20 (31 y), TD:19 (32 y)	Free Viewing Task with 700 Natural Scene Images (OSIE Dataset)	ASD: Higher Saliency Weights for Low Level Properties, Lower Weights for Object and Semantic-Based Properties
Voice	Min and Tewfik, 2010 [42]	Microphone	Audio, Accelerometer	ASD: 4	No Context Given	22/24 Vocal Stimming Events Detected by Classifier
Voice	Min and Fetzner, 2018 [43]	Microphone	Audio, 2D Imaging	ASD: 4	No Context Given	Trained Dictionaries Detect Vocal Stimming with Sensitivity: 73–93%
Voice	Marchi et al., 2015 [44]	Zoom H1 Handy Recorder (Hebrew, English), Zoom H4 With RØDE NTG-2 Microphone (Swedish)	Audio	ASD: 7 (Hebrew), 11 (Swedish), 9 (English); TD:10 (Hebrew), 9 (Swedish), 9 (English)	Repeating Sentences From 9 Emotional Stories	ASD: Generally Perform Poorer, In English And Swedish Angry Was Poorly Performed, In Hebrew Afraid Was Poorly Performed
Voice	Ringeval et al., 2010 [45]	Logitech USB Desktop Microphone	Audio	AD: 12 (10 y), PDD-NOS: 10 (10 y), SLI: 13 (10 y), TD: 73 (10 y)	Reading 26 Sentences with Certain Prosodic Dependencies (Descending, Falling, Floating, Rising)	AD: Intonation for Falling Floating and Especially Rising Was Worse Compared to TD
Body Movement	Gonçalves et al., 2014 [46]	Microsoft Kinect	Color Depth Sensors, IR Emitter, Microphone	ASD: 5 (9 y)	playing session with robot	Good Detection of Hand Flapping with DTW, But Susceptible to Noise
Body Movement	Jazouli et al., 2019 [47]	Microsoft Kinect V1	Color Sensor, IR Depth Sensors, IR Emitter, Microphone	ASD: 5 (5–10 y), TD: 5 (Training Data)	No Context Given	94% Overall Recognition Rate For Stereotyped Gesture Recognition ($P Algorithm)
Body Movement	Rynkiewicz et al., 2016 [47]	Microsoft Kinect	Color Sensor, IR Depth Sensors, IR Emitter	ASD: 33 (5–10 y)	ADOS-2-Tasks (Cartoon Task, Demonstration Task)	ASD: Females Present Better Non-Verbal Skills (Gestures), Although Communication Skills Were Lower
Body Movement	Anzulewicz et al., 2016 [48]	iPad	Touch Sensor; Accelerometer	ASD: 37 (4 y); TD: 45 (5 y)	Playing 2 Serious Games (Sharing, Coloring)	Differences in Pressure Going into the Device as Well as Differences in Gesture Kinematics and Form
Multimodal	Samad et al., 2017 [49]	Sony EVI-D70, Mirametrix S2	2D Imaging, Eye Tracker	ASD: 8 (13 y); TD: 8 (16 y)	Emotion Recognition and Manipulation Task Of 3D Faces	ASD: Have Uncontrolled Manifestation of FAU 12, Spontaneous Facial Responses Are Not Synchronized with Their Visual Engagement with Facial Expressions, Poor Correlation in Dynamic Eye-Hand- Movements
Multimodal	Jaiswal et al., 2017 [50]	Microsoft Kinect V2	Color Sensor, IR Depth Sensors, IR Emitter, Microphone	ASD: 22; ADHD: 4, ASD + ADHD: 11, TD: 18	Read and Listen to A Set Of 12 Short Stories (From ‘Strange Stories’ Task), Accompanied By 2–3 Questions	Classifier sensitivity for Control vs. Clinical Condition: 96.4%; ASD+ADHD vs. ASD: 93,9%
Social Behavior	Westeyn et al., 2012 [51]	BlueSense, Video Cameras	Touch Sensors, Motion Sensor, Microphone, 2D Imaging	High-Risk: 1, TD: 10 Adults; 12 Children	Playing with Smart Toys with Scripted Play Prompts	Retrieval Score of 59% for a Single Child Using Models Constructed from Adult Play Data
Social Behavior	Anzalone et al., 2014 [52]	Microsoft Kinect; Nao	Color Sensor, IR Depth Sensors, IR Emitter, Microphone	ASD: 16v (9 y); TD: 16 (8 y)	Nao Prompts JA by Gazing/By Gazing and Pointing/By Gazing, Pointing and Vocalizing at Pictures	ASD: Trunk Position Showed Less Stability in 4D Compared to TD Controls, Gazing Exploration Showed Less Accuracy
Social Behavior	Campbell et al., 2019 [53]	iPad	2D Imaging Sensor (Front Camera)	ASD: 22 (2 y); TD: 82 (2 y)	Reaction to Name-Calling While Watching Videos	ASD: Classifying by Atypical Orientation: Sensitivity: 96%, Specificity: 38%
Social Behavior	Petric et al., 2014 [54]	Nao Robot	Cameras, Microphones, Ultrasound Range Sensors, Tactile Sensors, Force Sensitive Resistors, Accelerometers	ASD: 3 (5–8 y), TD: 1 (6 y)	ADOS-Tasks (Name Calling, JA, Play Request, Imitation)	Descriptively High Correspondence of Human Rater and Algorithm

Note: Age in sample description typically refers to mean age of participants per group, with the exception of a few studies which report either age ranges, individual age of single cases, or did not specify exact age.

**Table 2:** Original Articles on Sensor-Based Supporting Interventions in ASD.

Domain of Behavior	Reference	Solution Name	Sensor/Parameters	Sample (Mean Age)	Setting and Stimuli	Results
Emotion	Gordon et al., 2014 [68]	Webcam	2D Imaging Sensor	ASD: 30 (11 y), TD: 23 (11 y)	Playing Computer Game (FaceMaze)	ASD: Increase in Happy and Angry Expression Performance
Emotion	Leo et al., 2015 [69]	Camera, Robokind™ R25 Robot	2D Imaging Sensor	ASD: 3	Imitate Expression from Robot (Happiness, Sadness, Anger, And Fear)	31/60 Interactions Recognized; 19/60 No Imitation
Emotion	Piana et al., 2019 [70]	Microsoft Kinect V2	Color Sensor, IR depth Sensors, IR Emitter	ASD: 10 (10 y)	10 Sessions Body Emotion Expression and Recognition Task	Increased Accuracy in Expression and Recognition After Training Sessions In The Trained Group, Transfer Effect On Facial Expression Recognition
Social Skills	Robins et al., 2010 [71]	KASPAR, Video Cameras	Touch Sensor, 2D Imaging	ASD: 3	Unconstrained Interaction with The Robot	Interaction Evaluation Through Sensor Activation, Differential Interaction and Maintenance of Interaction
Social Skills	Costa et al., 2009 [73]	LEGO Mindstorms TM, Video Cameras	Touch Sensor, Sound Sensor	ASD: 2 (17, 19 y)	4- 5 Sessions of Feedbacked Interaction with Robot	Interaction Evaluation Through Sensor Activation, Differential Interaction and Maintenance of Interaction
Social Skills	Wong and Zhong, 2016 [74]	CuDDler Robot, Video Camera	Microphone, Contact Microphone, Tactile and Posture Sensors	ASD: 8 (5 y)	5 Sessions of ABA (Didactic Teaching Followed by Role Modeling by Either A Robot (RT) Or Human (CT)	Robot Training Significantly Facilitated Verbal and Gestural Communicative Skills, Increased Eye Contact Duration
Social Skills	Uzuegbunam et al., 2015 [75]	Microsoft Kinect	Color Sensor, IR Depth Sensors, IR Emitter, Microphone	ASD: 3 (7–12 y)	Greeting Game with Participants Face, Reacting to Participant, Getting Appraisal	All 3 showed Increased Social Greeting Behavior Throughout and After Intervention
Social Skills	Mower et al., 2011 [76]	HDR-SR12 High Definition Handycam Camcorders	Microphones, 2D Video Sensors	ASD: 2 (6, 12 y)	4 Sessions with Embodied Conversational Agent RACHEL Going Through Emotional Scenarios	Tool for Eliciting Interactive Behavior

Note: Age in sample description, where specified in a study, either refer to mean age of participants per group, to age ranges, or to individual ages in small samples of single cases.
